# Delphinidin Inhibits Tumor Growth by Acting on VEGF Signalling in Endothelial Cells

**DOI:** 10.1371/journal.pone.0145291

**Published:** 2015-12-22

**Authors:** Thérèse Keravis, Laure Favot, Abdurrazag A. Abusnina, Anita Anton, Hélène Justiniano, Raffaella Soleti, Eid Alabed Alibrahim, Gilles Simard, Ramaroson Andriantsitohaina, Claire Lugnier

**Affiliations:** 1 Laboratoire de Biophotonique et de Pharmacologie, CNRS UMR 7213, Université de Strasbourg, Illkirch, France; 2 LUNAM, INSERM, U1063, Université d'Angers, Angers, France; 3 Centre Hospitalo-Universitaire, Angers, France; Columbia University, UNITED STATES

## Abstract

The vasculoprotective properties of delphinidin are driven mainly by its action on endothelial cells. Moreover, delphinidin displays anti-angiogenic properties in both *in vitro* and *in vivo* angiogenesis models and thereby might prevent the development of tumors associated with excessive vascularization. This study was aimed to test the effect of delphinidin on melanoma-induced tumor growth with emphasis on its molecular mechanism on endothelial cells. Delphinidin treatment significantly decreased *in vivo* tumor growth induced by B16-F10 melanoma cell xenograft in mice. *In vitro*, delphinidin was not able to inhibit VEGFR2-mediated B16-F10 melanoma cell proliferation but it specifically reduced basal and VEGFR2-mediated endothelial cell proliferation. The anti-proliferative effect of delphinidin was reversed either by the MEK1/2 MAP kinase inhibitor, U-0126, or the PI3K inhibitor, LY-294002. VEGF-induced proliferation was reduced either by U-0126 or LY-294002. Under these conditions, delphinidin failed to decrease further endothelial cell proliferation. Delphinidin prevented VEGF-induced phosphorylation of ERK1/2 and p38 MAPK and decreased the expression of the transcription factors, CREB and ATF1. Finally, delphinidin was more potent in inhibiting *in vitro* cyclic nucleotide phosphodiesterases (PDEs), PDE1 and PDE2, compared to PDE3-PDE5. Altogether delphinidin reduced tumor growth of melanoma cell *in vivo* by acting specifically on endothelial cell proliferation. The mechanism implies an association between inhibition of VEGF-induced proliferation via VEGFR2 signalling, MAPK, PI3K and at transcription level on CREB/ATF1 factors, and the inhibition of PDE2. In conjunction with our previous studies, we demonstrate that delphinidin is a promising compound to prevent pathologies associated with generation of vascular network in tumorigenesis.

## Introduction

Epidemiologic studies have shown that a diet rich in fruits and vegetables has a beneficial preventive effect for cardiovascular diseases and cancer [1–3]. We previously reported that the anthocyanin delphinidin possesses the same pharmacological profile as a total extract of red wine polyphenolic compounds to promote endothelial nitric oxide (NO) production via the activation of oestrogen receptor alpha [4,5], the increase of intracellular calcium concentration and activation of tyrosine kinases [6]. In addition, we have demonstrated that delphinidin displays anti-angiogenic properties in both *in vitro* and *in vivo* angiogenesis models [7–10]. Delphinidin acts on different steps leading to neovascularization including migration and proliferation induced by vascular endothelium growth factor (VEGF) in endothelial cells. The mechanisms involved include activation of ERK1/2, cyclin dependent-pathway and inhibition of VEGF-induced mitochondrial biogenesis [7–10] and inhibition of VEGF receptor 2 (VEGFR2) trans-activation [11]. Angiogenesis represents an essential step in tumor development and metastasis [12]. Then, during the vascular stage, tumor nutrition through diffusion is no longer sufficient and formation of new vasculature is necessary for tumor growth. Anti-angiogenic agents are now considered as an important cancer therapy option.

Based on the anti-angiogenic property of delphindin, the purpose of the present study was first, to investigate the effect of delphinidin on *in vivo* tumor growth induced by B16-F10 melanoma cell xenograft in mice, to characterize the respective contribution of melanoma and endothelial cells in delphinidin effects and consequently to decipher how delphinidin interacts with the intracellular signalling pathways activated by VEGF in endothelial cells with respect to proliferation.

## Materials and Methods

### Drugs and chemicals

Delphinidin (chloride form) was purchased from Extrasynthese (Genay, France) and TransMIT (Marburg, Germany). Human serum (HS) was from PromoCell and DMEM was from ATCC. All other cell culture products were from Lonza. Hybond-P polyvinylidene fluoride (PVDF) membranes, enhanced chemiluminescence (ECL) assay kit, and autoradiography films were from GE Healthcare. Horseradish peroxidase (HRP)-conjugated secondary antibodies were from Promega. The Reblot^TM^ plus kit from Chemicon was used for stripping membranes. The primary antibodies for total and phosphorylated ERK1/2 were from Sigma: anti-MAP Kinase-ERK1/2 (#M5670); anti-activated MAP Kinase-diphosphorylated (Thr^183^ and Tyr^185^ in ERK-2) ERK1/2 (#M8159). The primary antibodies for total and phosphorylated p38 MAP kinase were from Cell Signaling: anti-p38 MAPK (#9212); anti-phospho (Thr180/Tyr182); p38 MAPK (#9211). The primary antibodies for total and phosphorylated CREB were from Cell Signaling: phospho-CREB (Ser133) antibody (#9191) and CREB antibody (#9192). The phospho-CREB antibody was also used to detect the phosphorylated ATF1 (Ser63). All other Western blotting reagents were from Sigma. U-0126 [1,4-diamino-2,3-dicyano-1,4-bis(2-aminophenylthio)-butadiene] was used as MEK1/2 MAP kinase inhibitor [13] and was from Biomol. LY-294002 [2-(4-Morpholinyl)-8-phenyl-4H-1-benzopyran-4-one] was used as PI3 kinase inhibitor [14] and was from Euromedex. Recombinant human VEGF was from Cell Concepts. The primary antibodies for total and phosphorylated VEGFR2 were from (Cell signaling Technology). Ki8751, the specific inhibitor of VEGFR2 tyrosine kinase [15], was purchased from Santa Cruz Biotechnology.

Delphinidin was used in ethanol for tumor experiments and in dimethylsulfoxide (DMSO) for cell proliferation and protein expression experiments. U-0126 and LY-294002 were prepared in DMSO. The final concentration of DMSO in experiments never exceeded 0.1%. Control groups received the vehicle alone.

### Cells and animals

B16-F10 (ATCC^®^ CRL-6475 ^™^, C57BL/6J strain, batch # 3225557) mouse melanoma cells were purchased on March 2005 from American Type Culture Collection (LGC, Promochem, Molsheim, France). Primary human umbilical vein endothelial cells (HUVECs) were prepared as previously described [16], by collagenase digestion of freshly delivered umbilical cords obtained after written consent obtained by the “Service de Maternité” in the “Centre Hospitalo-Universitaire de Hautepierre de Strasbourg” (www.chru-strasbourg.fr/poles/Gynecologie-obstetrique) according to the principles expressed in the Declaration of Helsinki.

C57BL/6N mice (6 week old male, 20 g of weight) were obtained from Charles River. Mice were maintained in controlled temperature room (25°C) with a 12 hour-light/dark cycle and were provided with food and water ad libitum. This investigation was carried out in accordance with the Guide for the Care and Use of Laboratory Animals (National Institutes of Health Publication no. 85–23, revised 1996) and received authorization from the “*Comité Régional d'Ethique en Matière d'Expérimentation Animale de Strasbourg” President*: *Fabielle Angel-Urban*, *Institut de Physiologie Faculté de Médecine*, *Université Louis Pasteur*, *11 rue Humann 67085 Strasbourg (CEEA 35*, *approved by the Ministère de l’Enseignement Supérieur et de la Recherche* (CREMEAS, number: AL/02/06/05/09) http://www-ulp.u-strasbg.fr/cremeas/. The ethics committee of the University of Strasbourg specifically approved this study as well as the consent procedure.

### Mouse xenografts and treatments

**Fig 1:**
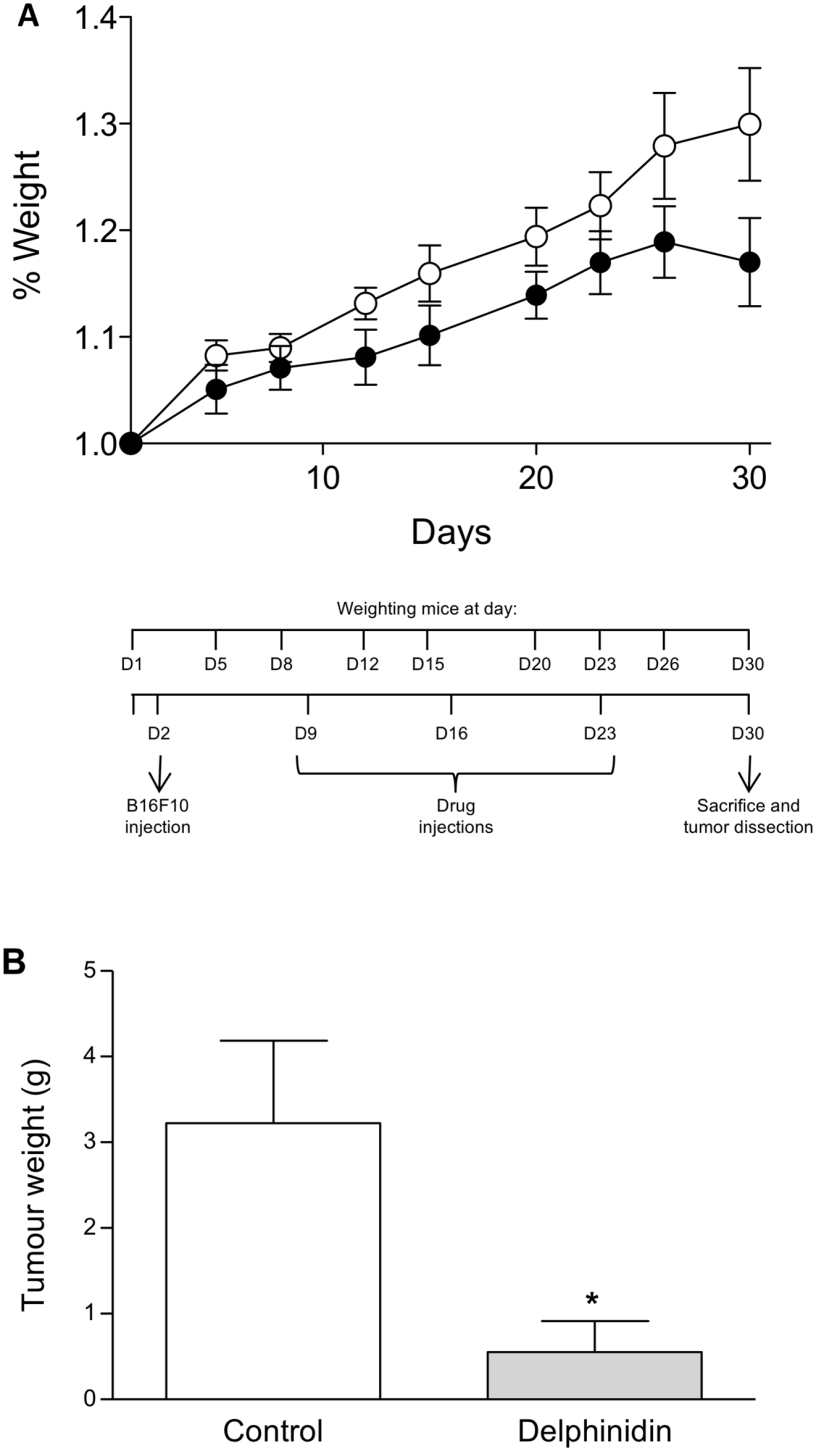
Effect of delphinidin on mouse tumor development. Mouse xenografts and treatments were conducted as described in Materials and Methods. (A) Experimental protocol and delphinidin effect on mouse weight. Mice were receiving delphinidin (●) or the vehicle of delphinidin as control (○). (B) Effect of delphinidin on tumor weight. On the 30^th^ day, mice were sacrificed and tumors were dissected. In comparison with control: * (*P* < 0.05).

Two groups of 7 male C57BL/6N mice were used at 6 weeks old for tumor induction. The experimental protocol is illustrated on in Fig 1A. Before any treatments, mouse weights were registered on day 1 (D1). On the following day (D2), all mice were subcutaneously injected on the ventral side near each hindleg with B16-F10 melanoma cells (25x10^4^ cells in 100 μl of 0.9% NaCl). The seventh day after tumor cell inoculation (D9), mice were treated by i.p. injection (100 μl/10 g mice) with either drug solvent (1% ethanol) or 10 mg delphinidin/kg. Delphinidin as well as solvent injections were repeated twice at 7-day intervals (D16 and D23). Then, seven days later (D30) after pentobarbital anesthesia, mice were sacrificed by cervical dislocation and tumors were dissected and weighted. Mouse weights were registered over 30 days on D1, D5, D8, D12, D15, D20, D23, D26 and D30.

### Cell culture

B16-F10 cell line and HUVECs were cultured at 37°C in a humidified incubator (5% CO_2_).

The cell line B16-F10 was cultured in T-175 flasks in DMEM supplemented with 100 U/ ml penicillin, 100 μg/ ml streptomycin and 10% heat-inactivated foetal calf serum. Cells were harvested by trypsination, washed with 0.9% NaCl and resuspended to get a density of 25x10^4^ cells in 100 μl of 0.9% NaCl and used right away for the tumor growth experiments.

HUVECs were grown on plastic flasks coated with 60 mg/l type I collagen and cultured in M199/RPMI 1640 (50:50, v/v) containing 2 μmol/ml ultraglutamine I, 100 U/ ml penicillin, 100 μg/ ml streptomycin, 2.5 μg/ml fungizone and supplemented with 20% serum, i.e. 10% of human serum (HS) + 10% of heat-inactivated foetal calf serum (high serum culture medium) as described previously [16]. Cells were used at the second passage.

### Melanoma cell proliferation

B16-F10 (5,000 cells per well) were seeded on 96-multiwell plates in completed medium and allowed to attach overnight. The medium was then replaced and cells were treated with or without 10 ng/ml of VEGF, in presence or in absence of 10 μg/ml delphinidin. Proliferation was determined at 24 h, 48 h and 72 h by a fluorimetric assay using the CyQUANT Cell Proliferation Assay Kit (Molecular Probes, Eugene, OR). After growth medium removal, dye-binding solution was added into each microplate well and cells were incubated at 37°C for 45 minutes. The fluorescence levels were read on a fluorescent microplate reader (Synergy HT, Biotek, Winooski, VT) with filters for 485 nm excitation and 530 nm emission [17].

### Endothelial cell proliferation

HUVECs (12,000 cells per well) were seeded on 24-multiwell plates, or in the case of VEGFR2 studies HUVECs (10000 cells per well) were seeded on 96-multiwell plates, in high serum culture medium and allowed to attach for 4–5 h. The 20% serum medium was then replaced by 1% HS medium. After 24 h incubation, medium was removed and replaced with 500 μl /well or 280 μl/well (for VEGFR2 studies) of 1% HS medium containing tested substances. Cells were pre-treated with or without a MEK1/2 MAP kinase inhibitor (U-0126, 10 μM) or a PI3 kinase inhibitor (LY-294002, 10 μM), then treated with or without 10 ng/ml of VEGF, in presence or in absence of 10 μg/ml delphinidin. Cells were allowed to proliferate for 3 days and HUVEC proliferation was determined at 24 h, 48 h, and 72 h by a colorimetric assay using the ‘CellTiter 96 AQeous One Solution Cell Proliferation Assay’ from Promega [18].

Cells were pre-treated with or without a VEGFR2 specific inhibitor (Ki8751, 10 nM), then treated with or without 10 ng /ml in presence or absence of 10 μg/ml delphinidin. Cells were allowed to proliferate for 2 days and HUVECs proliferation was determined at 24 h and 48 h by a colorimetric assay using the ‘CellTiter 96 AQeous One Solution Cell proliferation Assay’ from Promega.

### VEGFR2 expression

HUVECs and B16-F10 cells were treated with delphinidin (10 μg/ml) for 3 h and then incubated without or with VEGF (10 ng/ml) for 30 min for VEGFR2 phosphorylation or 24 h for VEGFR2 expression. Then, the cells were homogenized, washed twice with PBS and trypsinized. The cells were lysed with RIPA buffer freshly in presence of protease inhibitor. Cells were allowed to lyse for 45 min on ice and centrifuged at 15000 g for 15 min at 4°C. The supernatant was removed, and protein concentrations in the supernatant were determined by protein assay.

The proteins (30 μg) were separated on 10% sodium dodecyl sulfate-polyacrylamide gel electrophoresis (SDS-PAGE) and transferred on the nitrocellulose membranes. The membranes were incubated with anti-VEGFR2 or anti-pVEGFR2. The anti-β-actin (Sigma-Aldrich) was used to visualize protein gel loading. The membranes were then incubated with appropriate HRP-conjugated secondary antibody. The protein-antibody complexes were detected by chemiluminescence using the Western Blotting Luminol reagent kit (Santa Cruz Biotechnology^®^).

### Signalling protein expression

HUVECs (9,000 cells per dish of 3.5 cm diameter) were seeded on petri dishes in high serum culture medium and allowed to attach for 4–5 h. The 20% serum medium was then replaced by 1% HS medium. After 24 h incubation, medium was removed and replaced with 1 ml/petri dish of 1% HS medium with or without 10 ng/ml of VEGF, in presence or in absence of 10 μg/ml delphinidin. After 30 min of treatment, cells were harvested directly in Laemmli sample buffer (50 mM Tris-HCl, 0.005% bromophenol blue, 5% glycerol, 1% SDS, 2.5% β-mercaptoethanol). Appropriate quantities of extracted proteins were resolved in a SDS-10% polyacrylamide gel and electro transferred onto PVDF membranes, as described previously [19]. Membranes were processed for immunoblotting with primary antibodies directed against phosphorylated ERK1/2 (1/10,000), phosphorylated p38 MAP kinase (1/1,000), phosphorylated CREB (1/1,000), and phosphorylated ATF1 (1/1,000). Immobilized antigens were detected by chemiluminescence using HRP-conjugates as secondary antibodies (1/60,000), an ECL assay kit and autoradiography films. Autoradiography signals were captured on a GeneGenius Bio Imaging System (Syngene) using the GeneSnap software and analyzed using the GeneTools software. After being stripped, membranes were re-probing with an antibody directed against either total ERK1/2 (1/10,000) or total p38 MAP kinase (1/1,000) or total CREB (1/1,000). Immobilized antigens were detected as described above.

### Assessment of delphinidin effect on purified PDEs

PDE1, PDE3, PDE4 and PDE5 were isolated by anion-exchange chromatography from bovine aortic smooth muscle cytosolic fraction as previously described [20]. PDE2 was isolated from human platelets [21]. Purified PDEs were stored as aliquots at -80°C until use. PDE activity was determined by a radio-enzymatic assay as described previously [20,22]. The concentration of delphinidin that produced 50% inhibition (IC_50_) of substrate hydrolysis (determined at 1μM cAMP or cGMP) was calculated by non-linear regression analysis (GraphPad Prism, San Diego, CA) of concentration-response curves performed with 1–300 μM of delphinidin and included 6 different concentrations of delphinidin.

### Statistical analysis

Results are expressed as mean ± SEM of n experiments. Student’s *t* test for unpaired data was used for statistical analysis, with *P* < 0.05 being considered significant.

## Results

### Tumor development

The effect of delphinidin on mouse tumor development is illustrated on Fig 1. The evolution over the 30 day experimentation of mouse weight as a percent of the starting weight of each mouse (Fig 1A) was unchanged by delphinidin treatment (10 mg/kg i.p. once a week for 3 weeks) in comparison with non-treated mice (*P* = 0.914). Interestingly, delphinidin markedly decreased significantly the tumor weight on the 30^th^ day of experimentation by 83% (*P* = 0.02), as shown on Fig 1B.

The cellular origin of the effect of delphinidin was further characterized both on melanoma and endothelial cell proliferation.

### Melanoma cell and endothelial cell proliferations

**Fig 2:**
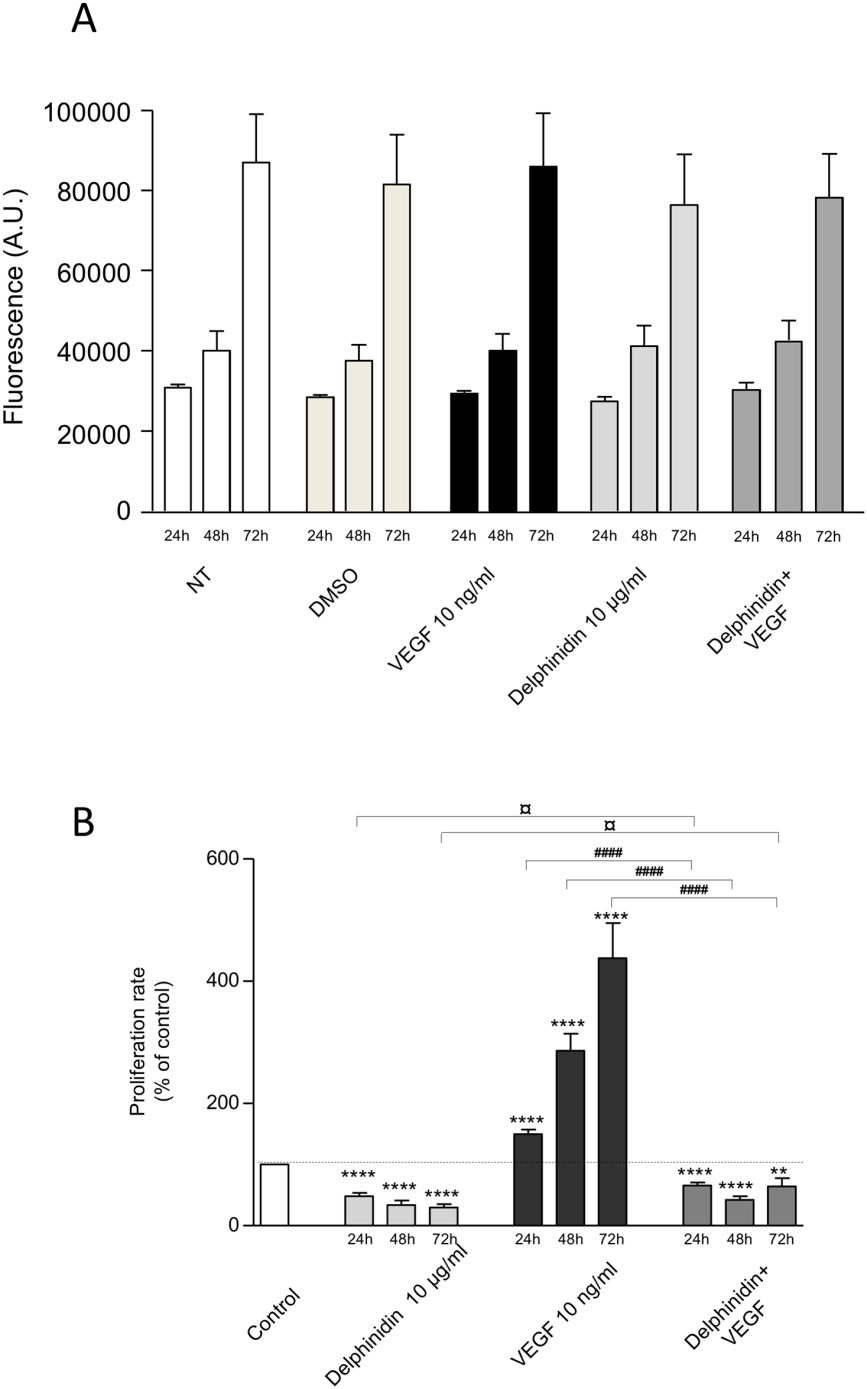
Effect of delphinidin and VEGF on melanoma cell (Fig 2A) and HUVEC proliferation (Fig 2B). (A) B16-F10 melanoma cells were incubated for 24, 48 and 72 h in cell culture medium containing 1% human serum with different additions. NT: non treated cells. DMSO: in presence of the solvent DMSO. and in presence of 10 μg/ml delphinidin, 10 ng/ml VEGF or 10 μg/ml delphinidin + 10 ng/ml VEGF. Cell number is expressed in arbitrary units. In each group n = 5. (B) HUVECs were incubated for 24, 48 and 72 h in cell culture medium containing 1% human serum, in absence of any additions (Control) and in presence of 10 μg/ml delphinidin, 10 ng/ml VEGF or 10 μg/ml delphinidin + 10 ng/ml VEGF. Cell proliferation rate is expressed in % of control (n = 12). In comparison with control: ** (*P* < 0.01), **** (*P* < 0.0001). In comparison with the respective incubation times—[Delphinidin+VEGF] versus Delphinidin: ¤ (*P* < 0.05)—[Delphinidin+VEGF] versus VEGF: #### (*P* < 0.0001).

As illustrated on Fig 2 (Fig 2A), neither delphinidin (10 μg/ml), nor VEGF (10 ng/ml) alone or in combination with delphinidin, were able to increase B16-F10 melanoma cell proliferation at any time tested (i.e. 24, 48 and 72 h).

In HUVECs, delphinidin (10 μg/ml) significantly decreased the rate of basal proliferation (-52% at 24 h, -66% at 48 h and -70% at 72 h, *P* < 0.0001 for each time point) (Fig 2B). As expected, VEGF (10 ng/ml) significantly increased the proliferation rate (+ 50% at 24 h, + 186% at 48 h and + 337% at 72 h, *P* < 0.0001 for each time-point). Interestingly, delphinidin completely prevented the ability of VEGF to induce endothelial cell proliferation at any time-point tested (-56% at 24 h, -85% at 48 h and -130% at 72 h; *P* < 0.0001 for each time point) and restored to some extent a proliferation rate close to the basal proliferation.

### Melanoma cell and endothelial VEGFR2-mediated cell proliferations

**Fig 3:**
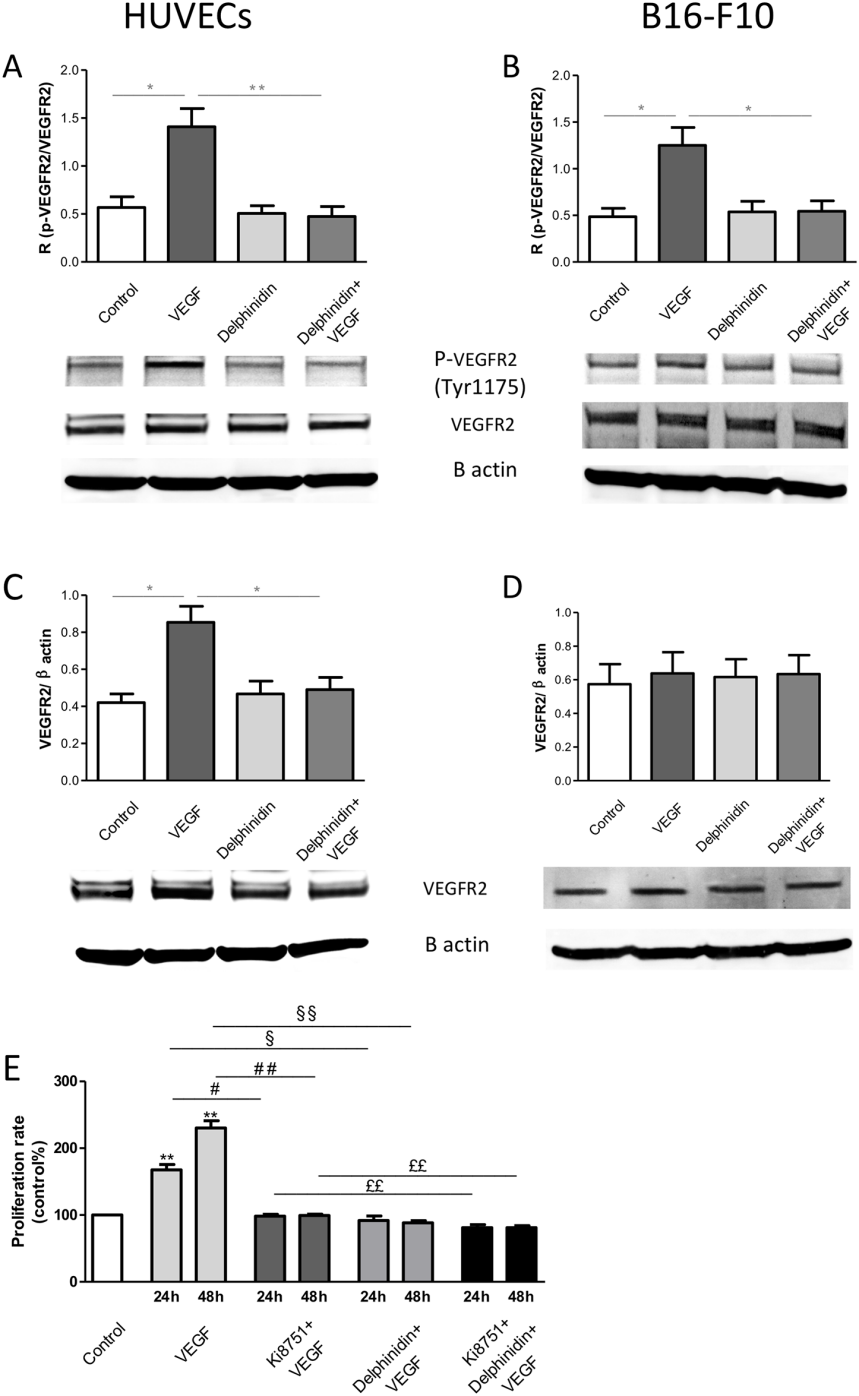
Effect of delphinidin and VEGF on VEGFR2. **VEGFR2 Phosphorylation**: (A) HUVECs and (B) B16-F10 were treated with delphinidin (10 μg/ml) for 3 h and then incubated without or with VEGF (10 ng/ml) for 30 min. HUVEC was cultured in M199/RPMI 1640 (50:50, v/v) containing 2 μmol/ml ultraglutamine I, 100 U/ ml penicillin, 100 μg/ ml streptomycin, 2.5 μg/ml fungizone and supplemented with 20% serum, i.e. 10% of human serum (HS) + 10% of heat-inactivated foetal calf serum (high serum culture medium) as described previously [16]. B16-F10 was cultured in DMEM supplemented with 100 U/ ml penicillin, 100 μg/ ml streptomycin and 10% heat-inactivated foetal calf serum. After treatment cells were harvested, cell lysates were prepared and the VEGFR2 expression and phosphorylation were determined. (A) and (B) represent the ratio of phosphorylated over total VEGFR2. Data are means ± SEM of n = 5 experiments. *P < 0.05 versus DMSO, ** *P* < 0.01 versus VEGF, ** *P* < 0.01 versus VEGF. **VEGFR2 expression**. (C) HUVECs and (D) B16-F10 were treated with delphinidin (10 μg/ml) for 3 h and then incubated without or with VEGF (10 ng/ml) for 30 min for VEGFR2 phosphorylation or 24 h for VEGFR2 expression. (A) and (B) represent the ratio of phosphorylated over total VEGFR2. Data are means ± SEM of n = 5 experiments. * *P* < 0.05 versus DMSO, ** *P* < 0.01 versus VEGF, ** *P* < 0.01 versus VEGF. **Effect of delphinidin, VEGF and Ki8751 on HUVECs proliferation** (E). HUVECs were incubated for 24 and 48 h in cell culture medium containing 1% human serum with different experimental conditions: Control, 10 ng/ml VEGF, 10 nM Ki8751 + 10 nM VEGF, 10 μg/ml delphinidin + 10 nM VEGF, 10 nM Ki8751 + 10 μg/ml delphinidin + 10 nM VEGF. Cell proliferation rate is expressed in % of control (n = 5). In comparison with control: *P < 0.05, ** *P* < 0.01. in comparison with the respective incubation times -[Ki8751 + VEGF] versus VEGF: # *P* < 0.05, ## *P* < 0.01 –[delphinidin+ VEGF] versus VEGF: § *P* < 0.05, §§ *P* < 0.01 –[Ki8751+delphinidin+VEGF] versus [Ki8751+VEGF]: ££ *P* < 0.01.

As expected, HUVECs express VEGFR2 (Fig 3) and VEGF increased both VEGFR2 expression (Fig 3A) and phosphorylation (Fig 3C). Interestingly, delphinidin alone had no effect but it completely prevented the ability of VEGF in increasing both VEGFR2 expression and phosphorylation. Moreover, the ability of VEGF to increase endothelial cell proliferation was completely abolished in the presence of the VEGFR2 specific inhibitor, Ki8751 (Fig 3E). Interestingly, delphinidin inhibited VEGF-induced proliferation and in this respect Ki8751 did not inhibit further this response. Altogether, these results demonstrate that delphinidin inhibits VEGF induced proliferation via the inhibition of VEGFR2 activation and expression.

In contrast to HUVECs, B16-F10 melanoma cells, although VEGF increased VEGFR2 phosphorylation after 30 min stimulation (Fig 3B), it did not modify VEGFR2 expression (Fig 3D). Since, VEGF was not able increase B16-F10 melanoma cells proliferation (Fig 2A), one can advanced the hypothesis that the ability of VEGF to enhance VEGFR2 phosphorylation was not sufficient on its own to activate all signalling pathways leading to proliferation. Taken together, these data reinforce our conclusion that the effect of delphinidin on melanoma-induced tumor growth in both *in vivo* and *in vitro* is mainly due to its action on endothelial cells. Therefore, the present study was focused on delphinidin participation in endothelial cell signalling.

### Proliferation and MEK-ERK-signalling pathway in endothelial cells

**Fig 4:**
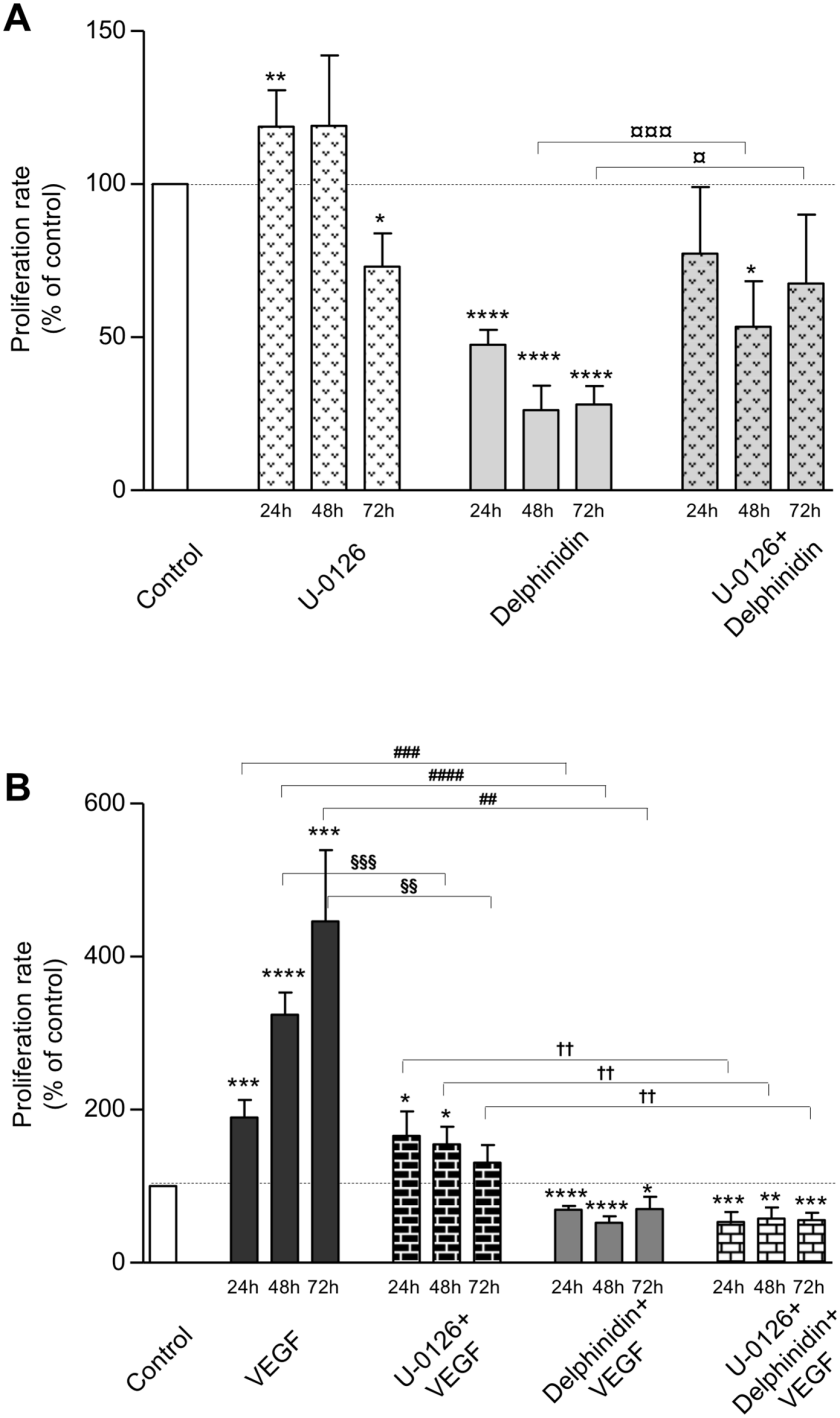
Effect of U-0126 and delphinidin on cellular proliferation. HUVECs were incubated for 24, 48 and 72 h in cell culture medium containing 1% human serum with different additions. (A) Basal proliferation: no addition (Control), in presence of 10 μM U-0126, 10 μg/ml delphinidin, and 10 μM U-0126 + 10 μg/ml delphinidin. (B) VEGF-stimulated proliferation: no addition (Control), 10 ng/ml VEGF, 10 μM U-0126 + 10 ng/ml VEGF, 10 μg/ml delphinidin + 10 ng/ml VEGF, 10 μM U-0126 + 10 μg/ml delphinidin + 10 ng/ml VEGF. Cell proliferation rate is expressed in % of control (n = 9). In comparison with control for (A) and (B): * (*P* < 0.05); ** (*P* < 0.01); *** (*P* < 0.001); **** (*P* < 0.0001). In comparison with the respective incubation times—[U-0126+Delphinidin] versus Delphinidin: ¤ (*P* < 0.05); ¤¤¤ (*P* < 0.001)—[U-0126+VEGF] versus VEGF: §§ (*P* < 0.01); §§§ (*P* < 0.001)—[Delphinidin+VEGF] versus VEGF: ## (*P* < 0.01); ### (0.001); #### (*P* < 0.001)—[U-0126+Delphinidin+VEGF] versus [U-0126+VEGF]: †† (*P* < 0.01).

Fig 4 shows the effect of U-0126 (10 μM) on basal (Fig 4A) and VEGF-stimulated proliferation in HUVECs (Fig 4B).

U-0126 (10 μM) slightly modified proliferation, although remaining near the basal level (+ 19% at 24 h, *P* = 0.009; + 19% at 48 h, ns; - 30% at 72 h, *P* = 0.02). However, *per se* delphinidin strongly inhibited basal rate proliferation. U-0126 reversed to some extent closer to basal level the inhibitory effect of delphinidin (Fig 4A).

U-0126 prevented to some extent the VEGF-induced proliferation (- 13% at 24 h, ns; - 52% at 48 h, *P* = 0.0004; - 70% at 72 h, *P* = 0.0022). Thus, the proliferation rate at 72 h was not significantly different from the control proliferation rate (131% at 72 h, n.s.). Interestingly, no further inhibition was observed when delphinidin was associated to U-0126, indicating that maximal inhibitory effect was already reached with delphinidin alone (Fig 4B).

### Proliferation and PI3 kinase-signalling pathway in endothelial cells

**Fig 5:**
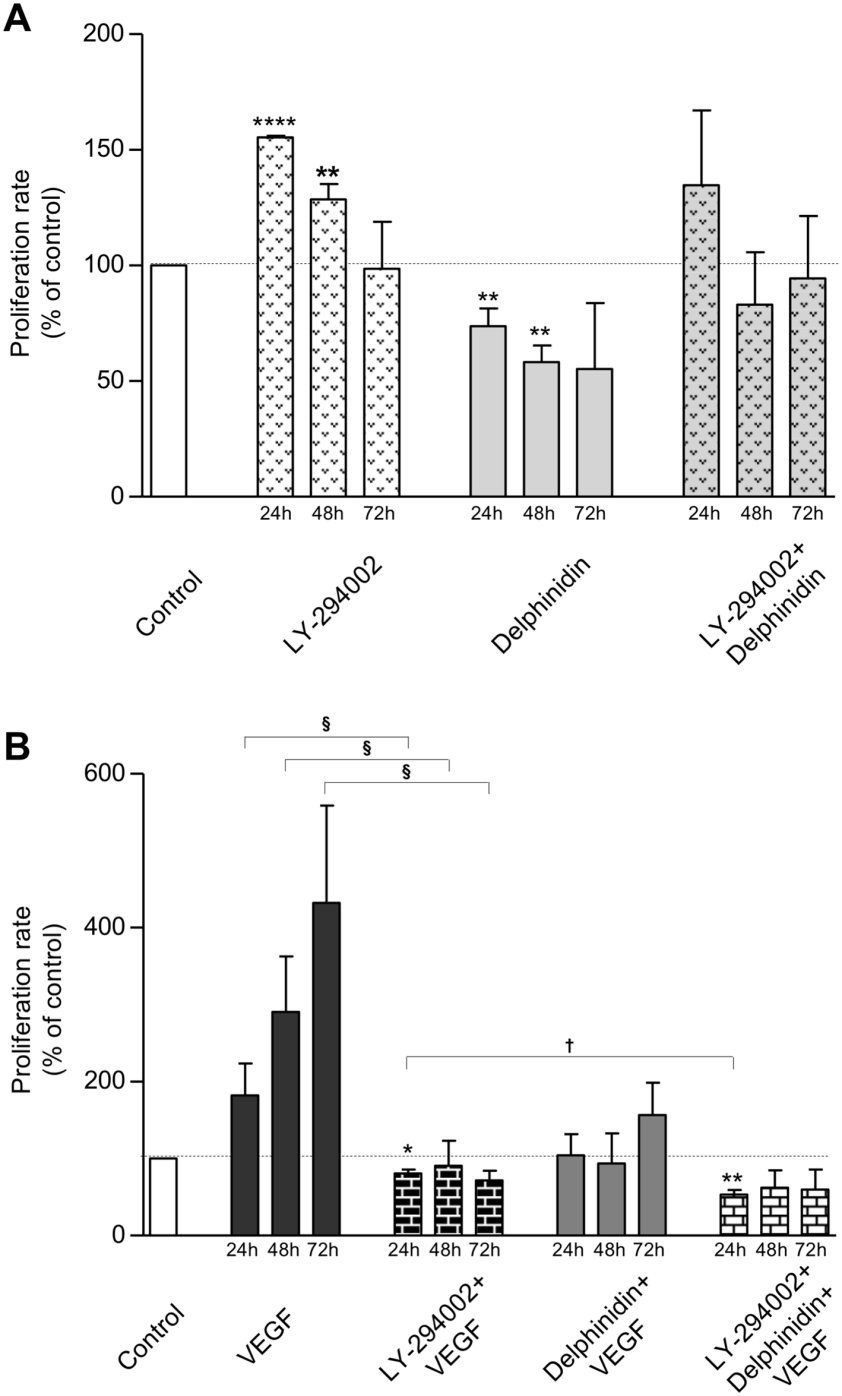
Effect of LY-294002 and delphinidin on cellular proliferation. HUVECs were incubated for 24, 48 and 72 h in cell culture medium containing 1% human serum with different additions. (A) Basal proliferation: no addition (Control), in presence of 10 μM LY-294002, 10 μg/ml delphinidin, and 10 μM LY-294002 + 10 μg/ml delphinidin. (B) VEGF-stimulated proliferation: no addition (Control), 10 ng/ml VEGF, 10 μM LY-294002 + 10 ng/ml VEGF, 10 μg/ml delphinidin + 10 ng/ml VEGF, 10 μM LY-294002 + 10 μg/ml delphinidin + 10 ng/ml VEGF. Cell proliferation rate is expressed in % of control (n = 3). In comparison with control for (A) and (B): * (*P*<0.05); ** (*P*<0.01); *** (*P*<0.001); **** (*P*<0.0001). In comparison with the respective incubation times–[LY-294002+VEGF] versus VEGF: § (*P* < 0.05)–[LY-294002+Delphinidin+VEGF] versus [LY-294002+VEGF]: † (*P* < 0.05).

Fig 5 shows the effect of LY-294002 (10 μM) on basal (Fig 5A) and VEGF-stimulated proliferation (Fig 5B).

LY-294002 significantly increased the basal proliferation at 24 h (+ 55%, *P* < 0.0001) and 48 h (+ 28%, *P* = 0.0068), but not at 72 h (Fig 5A). LY-294002 completely blunted the inhibitory effect of delphinidin.

LY-294002 abolished the ability of VEGF to increase proliferation at any time tested (- 44% at 24 h, *P* = 0.02; - 58% at 48 h, *P* = 0.03; - 83% at 72 h, *P* = 0.0001) (Fig 5B) and this, in absence or in presence of delphinidin.

### ERK1/2 and p38 MAP kinase in endothelial cells

**Fig 6:**
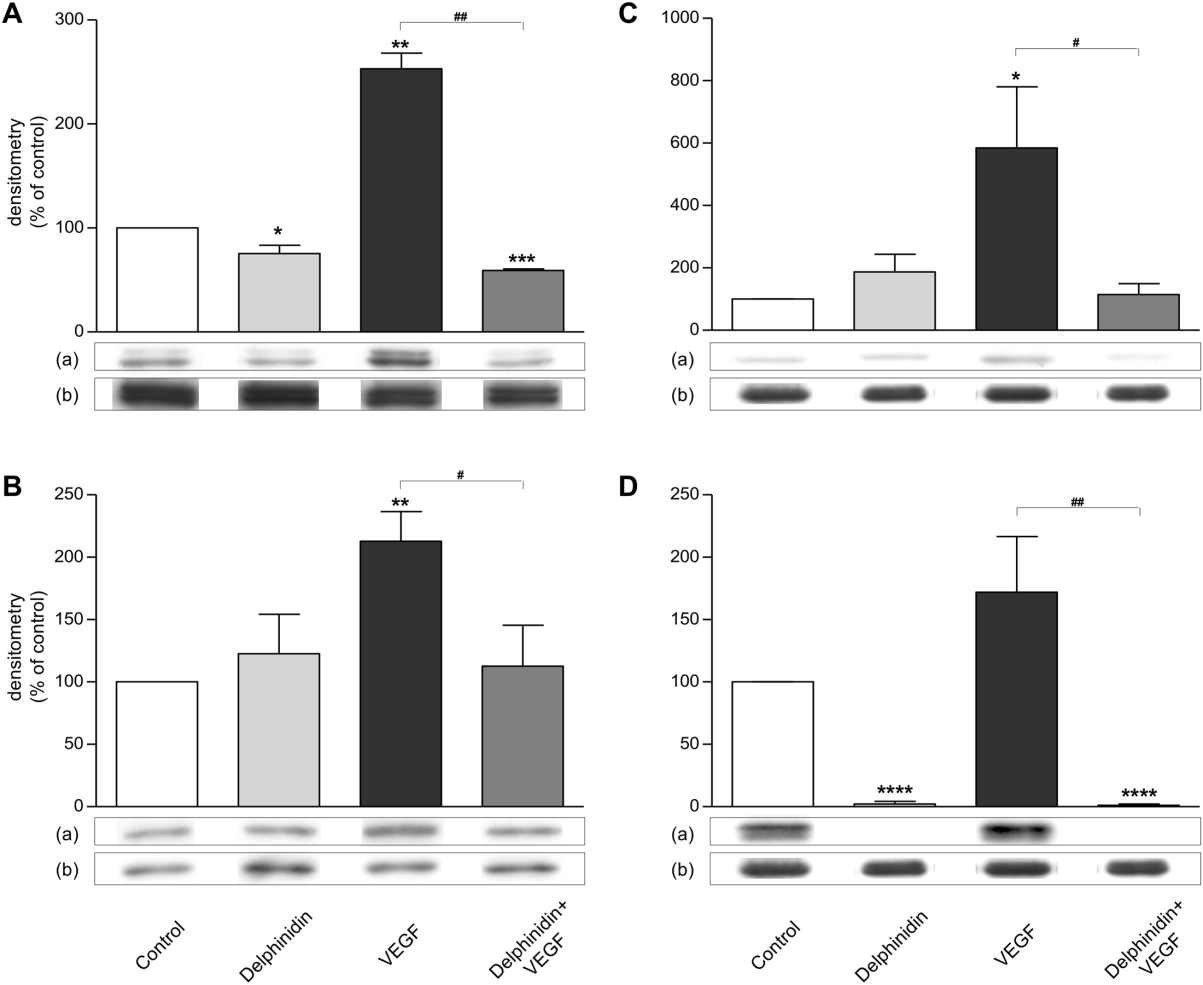
Effect of delphinidin and VEGF on ERK1/2 (Fig 6A), p38 MAP kinase activation (Fig 6B), CREB (Fig 6C) and ATF1 (Fig 6D) transcription factor activation. HUVECs were treated for 30 minutes with or without 10 μg/ml delphinidin, in absence or presence of 10 ng/ml VEGF. Analysis of 30 μg protein was performed in each condition. (A) Densitometry represents the ratio of phosphorylated (a) over total (b) ERK1/2 and is expressed in % of control (n = 4). In comparison with control: * (*P* < 0.05), ** (*P* < 0.01), *** (*P* < 0.001). In comparison with VEGF: ## (*P* < 0.01). (B) Densitometry represents the ratio of phosphorylated (a) over total (b) p38 and is expressed in % of control (n = 4). In comparison with control: ** (*P* < 0.01). In comparison with VEGF: # (*P* < 0.05). (C) Densitometry represents the ratio of phosphorylated (a) over total (b) CREB and is expressed in % of control (n = 4). In comparison with control: * (*P*<0.05). In comparison with VEGF: # (*P*<0.05). (D) Densitometry represents the ratio of phosphorylated ATF1 (a) over total CREB (b) and is expressed in % of control (n = 4). In comparison with control: **** (*P* < 0.0001). In comparison with VEGF: ## (*P* < 0.01).

Fig 6 shows that VEGF increased ERK1/2 activation (Fig 6A; + 153%, *P* = 0.005) and p38 MAP kinase activation (Fig 6B; + 113%, *P* = 0.002). Delphinidin decreased slightly but significantly basal ERK1/2 activation (- 25%, *P* = 0.0449) and abolished the ability of VEGF to activate this MAP kinase (- 77%, *P* = 0.0030) (Fig 6A).

Delphinidin did not affect basal p38 MAP kinase activation, but it completely prevented the activation of this kinase by VEGF (- 47%, *P* = 0.0246) (Fig 6B).

### CREB and ATF1 in endothelial cells

Fig 6 shows that delphinidin alone had no significant effect but it completely prevented the increase of CREB phosphorylation (+484%, *P* = 0.02) induced by VEGF (Fig 6C). Delphinidin abolished the phosphorylation of ATF1 in the control conditions (*P* < 0.0001) as well as after stimulation by VEGF (*P* = 0.0043) (Fig 6D).

### Effect on purified PDE activities

PDEs play a critical role in intracellular cAMP and cGMP signalling [23]. We have previously shown that different PDE families participate specifically in cell proliferation and migration as well as in VEGF-induced angiogenesis [24,25]. Also, the present study investigated the effect and the specificity of delphinidin towards PDE1 to PDE5 activities [26]. As illustrated on Table 1, delphinidin inhibited the activity of PDE1 to PDE5 in the range of 5 to 40 μg/ml concentrations, in agreement with its effect on cellular proliferation. Interestingly, delphinidin preferentially inhibited at similar concentrations the hydrolysis of cGMP by CaM/Ca-activated PDE1 (IC_50_ value of 5 μM) and the hydrolysis of cAMP by cGMP-activated PDE2 (IC_50_ value of 6 μM).

**Table 1:** IC_50_ values of delphinidin on purified PDE1 to PDE5 (μg/ml, μM).

	PDE1		PDE2		PDE3	PDE4	PDE5
Substrate	cGMP	cGMP	cAMP	cAMP	cAMP	cAMP	cGMP
Regulator		+CaM/Ca		+5μM cGMP			
μg/ml	11±2	5±1	35±3	6±1	12±2	37±4	15±2
μM	33±6	15±3	103±9	18±3	35±6	109±12	44±6

These data were preliminarily reported [26] and represented the mean of 3 determinations ± S.E.M.

## Discussion

Angiogenesis is critical for tumor development, and neovascularization is now known as a pre-requisite to the rapid expansion of tumor cells associated with formation of macroscopic tumors. Based on our previous data showing that delphinindin displays anti-angiogenic properties both *in vitro* and *in vivo*, its effect on tumor growth induced by B16-F10 melanoma cell xenograft was investigated in mice. The results suggest that delphinidin markedly reduces the increase of tumor growth *in vivo* by its action on endothelial but not on B16-F10 melanoma cell proliferation. The mechanism implies an association between the inhibition of VEGF-induced proliferation via MAPK and PI3K, the inhibition at transcription level on CREB/ATF1 factors, and the inhibition of PDE2 in endothelial cells.

*In vivo* delphinidin treatment reduced tumor growth induced by B16-F10 melanoma cell xenograft. It decreased tumor weight by 83% on the 30^th^ day of experimentation. Interestingly, no significant weight loss (%) was observed along the treatment indicating that delphinidin treatment did not induce adverse effect leading to weight loss. Moreover, these data reinforce the hypothesis that delphinidin acts specifically in reduced tumor growth weight. Interestingly, these data are strengthened by very recent data obtained for tumor growth induced by non-small-cell lung cancer cells in nude mice (NSCLC) [27]. Indeed, these authors have found that in athymic nude mice subcutaneously implanted with human NSCLC cells, delphinidin treatment causes: (i) a significant inhibition of tumor growth, (ii) a significant decrease in the expression of markers for cell proliferation (Ki67 and PCNA) and angiogenesis (CD31 and VEGF), and (iii) a significant induction of apoptosis, when compared with control mice.

To go any further in the molecular mechanism of delphinidin in the inhibition of tumor growth, we investigated *in vitro* on one hand the effect of delphinidin on B16-F10 melanoma cell proliferation linked to tumor induction and on the other hand on endothelial cell proliferation directly linked to angiogenesis.

Surprisingly, we found that delphinidin did not overcome basal- and VEGF-induced melanoma cell proliferation whereas interestingly delphinidin over 72 h of treatment significantly and potently inhibited basal and VEGF-induced endothelial cell proliferation as previously shown [8]. To go further, the expression and the phosphorylation of VEGFR2 receptor was studied in both endothelial and melanoma cells. Furthermore the compound Ki8751, which selectively antagonizes VEGFR2 receptor [28], was used for studying VEGF-induced cell proliferation in endothelial cells. The data clearly show that both cells express VEGFR2 which VEGF-dependent phosphorylation is inhibited by delphinidin treatment at the early stage of stimulation (i.e. 30 min) at which no change in VEGFR2 expression was observed yet. In contrast, VEGFR2 expression was increased after 24 h of incubation. Under these experimental conditions, delphinidin completely prevented the increase of VEGFR2 expression in HUVECs but not in B16-F10 cells. Taken together, these data reinforce our conclusion that the effect of delphinidin on melanoma-induced tumor growth in both *in vivo* and *in vitro* is mainly due to its action on endothelial cells and probably VEGFR2 signalling participates to its action.

These results suggest that the main target of delphinidin in reducing several angiogenic factors, including tumor growth, is due to its action on endothelium-dependent angiogenesis, although one cannot exclude that it might affect B16-F10 melanoma cell proliferation *in vivo*. Indeed, one study shows that oral consumption of delphinidin delays tumor growth in a lung carcinoma xenografted model through an inhibition of PDGF ligand/receptor signalling. This inhibitory effect of delphinidin on PDGF-R leads to the inhibition of PDGF-induced activation of ERK-1/2 signalling [29].

VEGF significantly increased HUVEC proliferation up to 72 h. The time-dependent study showed that this increase was already significant at 24 h in agreement with its 24 h-effect on cell cycle progression [8] and increased with time. Delphinidin treatment *per se* decreased significantly and potently basal and VEGF-induced proliferation rate (stabilized at 72 h treatment) suggesting that it acts on canonical pathways implicated in basal as well as VEGF-activated intracellular signalling. VEGF via VEGF-R2 is the most important pathway in inducing endothelial cell proliferation, migration and tube formation leading to angiogenesis. Thus, delphinidin exerts its anti-angiogenic activity, at least in part, by inhibiting VEGF-R2 activation. VEGF signalling is mediated by activation of extracellular signal-regulated kinase ERK1/2 [30] and via Akt activation [31]. Furthermore, signalling by the PI3-AKT [32] and MEK1/2-ERK1/2 pathways can collaborate to maintain cell viability [33].

In the present study, delphinidin reduced basal proliferation by a mechanism sensitive both to ERK1/2 inhibitor in association with diminished ERK1/2 phosphorylation and to PI3K/Akt inhibitor. In conjunction with these results, we have previously reported that delphinidin in basal condition displays anti-proliferative effect through an ERK1/2 phosphorylation, independent of nitric oxide pathway and this effect is correlated with suppression of cell progression by blocking the cell cycle in G0/G1 phase in bovine aortic endothelial cells [8]. According to a mouse Matrigel plug assay, the anti-angiogenic properties of delphinidin is mediated *via* an inhibition of vessel formation by basic FGF-2 and PDGF [29]. Serum used in the present study contains several growth factors including PDGF. Thus, one can advance the hypothesis that the inhibitory effect of delphinidin on basal proliferation might be due to its effect on PDGF signalling. Altogether, delphinidin affects basal endothelial proliferation by acting on ERK1/2 and PI3K/Akt.

The most important findings of the present study were that delphinidin was able to prevent VEGF-induced proliferation and activation of both ERK 1/2 and p38 MAP kinase by a mechanism sensitive to PI3/Akt inhibitors. These data strengthened our previous studies showing that delphinidin *in vitro* prevents VEGF-induced increase of mitochondrial biogenesis and activation of Akt pathway [10] and delphinidin *in vivo* reduces angiogenesis similarly to that obtained with high dose of red wine polyphenols via an inhibition of NO/VEGF and Akt/PI3K pathways [9]. In the later study, MMP activity is also reduced by delphinidin in association with p38 MAP kinase and NF-kappaB expression. Altogether, these data are consistent with the fact that delphinidin might inhibit phosphorylation of VEGF-R2 and consequently diminish multiple signalling pathways activated by VEGF in endothelial cells in a similar fashion than that reported by Lamy et al. 2006 [34].

Since inhibitor of p38 MAPK signalling is able to block the CREB phosphorylation in HUVEC [35], it was of interest to investigate the effect of delphinidin on the phosphorylation of CREB and ATF1 transcription factor [36] which are regulated by MAPK and PI3K signalling and participates to cAMP signalling. The present study underscores for the first time that delphinidin was able to overcome the VEGF-induced phosphorylation of both transcription factors, this effect being maximal on ATF1 phosphorylation. These phosphorylations are a requisite for their DNA binding [36]. Interestingly, in absence of VEGF delphinidin already inhibited ATF1 phosphorylation. This mechanism might explain and contribute to the delphinidin anti-proliferative effect on basal proliferation.

Finally, our previous studies have clearly demonstrated that among the cyclic nucleotide phosphodiesterase families (PDE1-PDE11; for review see [23] and [25]), PDE2 and PDE4 specifically control intracellular cAMP second messenger in HUVECs and participate to the control of VEGF-induced angiogenesis [16,24,37]. Notably, they control HUVEC migration, proliferation and cell cycle progression by modulating the expression of protein partners of VEGF signalling cascade, such as MAP kinase, cyclin A, cyclin D1, p21 and p27 [24,38]. Additively, we showed that polyphenols are able to differently inhibit PDE1 to PDE5 and to control vascular function [39,40]. In the present study delphinidin inhibited selectively the hydrolysis of cGMP by Ca^++^/CaM activated PDE1 and the hydrolysis of cAMP by cGMP-activated PDE2 in a concentration range that is in agreement with its effectiveness on tumor growth and *in vitro* HUVEC proliferation. Although delphinidin (10 μg/ml) did not inhibit *in vitro* B16-F10 proliferation, it might nevertheless partially explain the decrease of B16F10 tumor growth induced by delphinidin *in vivo*, since PDE1 inhibition *per se* reduces B16F10 melanoma cell proliferation [41]. Additively, delphinidin similarly to PDE2 inhibitor decreased VEGF-induced HUVEC proliferation [16]. Concerning the PDE2 contribution in VEGF induced HUVEC proliferation, it is of interest to know that: i) PDE2 might be activated by PMA-stimulated PKC [42]; ii) PDE2 is overexpressed in VEGF-stimulated HUVEC and its selective inhibition overcome HUVEC proliferation and migration [16,24,43]; iii) PMA markedly increased the expression of VEGF in HUVEC indicating that PKC is a key partner of VEGF signalling cascade [44]. Therefore, delphinidin might be helpful for the prevention and treatment of cancer, possibly by acting on PDE1 and PDE2, which regulate cyclic nucleotide levels.

In summary, delphinidin reduced tumor growth of melanoma cell *in vivo* by acting specifically on endothelial cell proliferation assessed by its selective effect towards endothelial cell VEGFR2 signalling. The mechanism implies an association between: (i) the inhibition of VEGF-induced proliferation via MAPK and PI3K; (ii) the inhibition at transcription level of the VEGF-induced phosphorylation of CREB/ATF1 factors; and (iii) the inhibition of PDE2. In conjunction with our previous study, we demonstrate that delphinidin is a promising compound to prevent pathologies associated with generation of vascular network in tumorigenesis.
